# Multicenter, phase II clinical trial of cancer vaccination for advanced esophageal cancer with three peptides derived from novel cancer-testis antigens

**DOI:** 10.1186/1479-5876-10-141

**Published:** 2012-07-09

**Authors:** Koji Kono, Hisae Iinuma, Yasunori Akutsu, Hiroaki Tanaka, Naoko Hayashi, Yasuto Uchikado, Tsuyoshi Noguchi, Hideki Fujii, Kota Okinaka, Ryoji Fukushima, Hisahiro Matsubara, Masaichi Ohira, Hideo Baba, Shoji Natsugoe, Seigou Kitano, Kazuyoshi Takeda, Koji Yoshida, Takuya Tsunoda, Yusuke Nakamura

**Affiliations:** 1 First Department of Surgery, University of Yamanashi, Yamanashi, Japan; 2 Department of Surgery, Teikyo University, Teikyo, Japan; 3 Department of Frontier Surgery, Graduate School of Medicine, Chiba University, Chiba, Japan; 4 First Department of Surgery, Osaka City University, Osaka, Japan; 5 Department of gastroenterological surgery, Kumamoto University, Kumamoto, Japan; 6 Department of digestive surgery, Kagoshima University, Kagoshima, Japan; 7 Department of gastroenterological surgery, Oita University, Oita, Japan; 8 Department of Immunology, Juntendo University School of Medicine, Juntendo, Japan; 9 Laboratory of Molecular Medicine, Human Genome Center, Institute of Medical Science, the University of Tokyo, Tokyo, Japan; 10 Department of Surgery, National University of Singapore, Level 8, NUHS Tower Block, NUHS 1E Kent Ridge Road, 119228, Singapore, Singapore

**Keywords:** Cancer vaccine, Esophageal cancer, Phase II clinical trial, CTL, Peptide vaccine

## Abstract

**Background:**

Since a phase I clinical trial using three HLA-A24-binding peptides from TTK protein kinase (TTK), lymphocyte antigen-6 complex locus K (LY6K), and insulin-like growth factor-II mRNA binding protein-3 (IMP3) had been shown to be promising for esophageal squamous cell carcinoma (ESCC), we further performed a multicenter, non-randomized phase II clinical trial.

**Patients and methods:**

Sixty ESCC patients were enrolled to evaluate OS, PFS, immunological response employing ELISPOT and pentamer assays. Each of the three peptides was administered with IFA weekly. All patients received the vaccination without knowing an HLA-A type, and the HLA types were key-opened at the analysis point. Hence, the endpoints were set to evaluate differences between HLA-A*2402-positive (24(+)) and -negative (24(−)) groups.

**Results:**

The OS in the 24 (+) group (n = 35) tended to be better than that in the 24(−) group (n = 25) (MST 4.6 vs. 2.6 month, respectively, *p* = 0.121), although the difference was not statistically significant. However, the PFS in the 24(+) group was significantly better than that in the 24(−) group (*p* = 0.032). In the 24(+) group, ELISPOT assay indicated that the LY6K-, TTK-, and IMP3-specific CTL responses were observed after the vaccination in 63%, 45%, and 60% of the 24(+) group, respectively. The patients having LY6K-, TTK-, and IMP3-specific CTL responses revealed the better OS than those not having CTL induction, respectively. The patients showing the CTL induction for multiple peptides have better clinical responses.

**Conclusions:**

The immune response induced by the vaccination could make the prognosis better for advanced ESCC patients.

**Trial registration:**

ClinicalTrials.gov, number NCT00995358

## Background

Recent approval of Sipuluecel-T, which is cancer vaccine with an activated antigen presenting cells and lymphocyte mixture for hormone refractory prostate cancer and anti-CTLA4 mAb (Ipilimimab) for melanoma implicate a new era of the immunotherapy in the anti-cancer strategy [1,2]. Therapeutic cancer vaccines against tumor-specific antigens have suggested the improvement of patient`s survivals [1-4]. Moreover, several phase III randomized trials of cancer vaccination with various tumor antigens against different types of tumor are on-going [5].

We previously reported that three novel HLA-A24-restricted immunodominant peptides, which were derived from three different Cancer-Testis antigens, TTK protein kinase (TTK), lymphocyte antigen 6 complex locus K (LY6K), and insulin-like growth factor (IGF)-II mRNA binding protein 3 (IMP-3), were promising targets for cancer vaccination for ESCC patients [6] because of their specific and frequent expression in tumor tissues [6-12]. Since these three genes were also shown to be essential for cancer cell growth/survival, a possibility of immune escape of cancer cells by losing protein expression is very low. Moreover, it has shown that these three peptides could stimulate CTLs that recognized and killed ESCC cells endogenously expressing these antigens [7] and that pre-existence of specific T cells, which could respond to the peptides derived from TTK, LY6K, and IMP3, were present in tumor-infiltrating lymphocytes (TIL), regional lymph node lymphocytes (RLNL), and PBLs obtained from HLA-A*2402 positive patients with ESCC [12]. Therefore, we had performed a phase I clinical cancer vaccination trials with a combination of multiple peptides that were derived from TTK, LY6K, and IMP3 for the HLA-A*2402 (+) patients with advanced ESCC who had been refractory to standard ESCC therapy, and the evidence in the phase I trial encouraged us to further develop this therapy as a phase II trial [6].

In the present report, we evaluated the survival benefit of the cancer vaccination and immunological monitoring in the phase II study for the patients with advanced ESCC who had been refractory to standard therapy.

## Materials and methods

### Study design

The present study is phase II, open-label, non-randomized, multicenter (7 centers in Japan) cancer vaccine trial as an exploratory setting. All enrolled patients had received the vaccination without knowing HLA-A status, and the HLA-A genotypes were used for key-open at analysis point. The endpoints were evaluated by differences between HLA-A*2402-positive (24(+)) and -negative (24(−)) groups as a biological marker for the sub-group analysis. Vaccination with a mixture of multiple peptides, that were derived from TTK, LY6K, and IMP3, and incomplete Freund’s adjuvant (IFA; Montanide ISA51, SEPPIC) was performed to the ESCC patients (n = 60) with locally advanced, recurrent, or/and metastatic tumors who had been resistant to standard therapy. HLA-A genotyping in all enrolled patients was performed by middle resolution genotyping method at HLA Laboratory (Kyoto, Japan).

The primary endpoint for evaluation in this study was overall survivals (OS). The secondary endpoint was progression free survivals (PFS), immunological responses, and adverse effects. Toxicities caused by the vaccination therapy were assessed by Common Terminology Criteria for Adverse Events version 3 (CTCAE). Immunological monitoring was performed at the central laboratory by both enzyme-linked immunospot (ELISPOT) assay and a pentamer assay using *in vitro* culturing lymphocytes derived from PBLs at pre- and post-vaccination periods, as described below. The OS, which was measured in days from the 1^st^ vaccination to death, was analyzed by the Kaplan-Meier method, and the PFS was measured in days from the1^st^ vaccination to disease progression.

We estimated that 29 patients in the 24 (+) group and 20 patients in the 24(−) group would be required by the assumption of a survival rate at 6 months of 30% in the 24(−) group and 45% in the 24(+) groups with overall alpha levels of 0.2 and beta levels of 0.5 as an exploratory setting. Considering the distribution (about 60%) of HLA-A*2402 in the Japanese population [13] and some drop-out cases, we decided to enroll a total of 60 patients.

The evaluation of endpoints was performed by intention-to-treat analysis. This study was approved by the institutional review board at each University (Approval Number at University of Yamanashi of Principal Investigator, No.484) and was registered with ClinicalTrials.gov, number NCT00995358. Written informed consent was obtained from all participants. The trial was carried out in accordance with the Helsinki declaration on experimentation on human subjects.

### Patient eligibility

The eligibility criteria of patients participating in the clinical trial were as follows. A) ESCC patients with locally advanced, recurrent, or/and metastatic tumors who had failed to respond to the standard therapy; B); Adequate bone-marrow, cardiac, pulmonary, hepatic, and renal functions including WBC of ≥2,000/mm^³^, platelet count of ≥75,000/mm^³^, total bilirubin of ≤2.0 of the institutional normal upper limit, AST, ALT, ALP of less than 2.5 times of the institutional normal upper limits, creatinine of ≤1.5 of the institutional normal upper limit; C) No therapy in 4 weeks prior to the initiation of the trial: D) ECOG performance status of 0–2; and E) age of 20–80 years old. The exclusion criteria of patients participating in the clinical trial were as follows. A) Pregnancy (women of childbearing potential: refusal or inability to use effective means of contraception); B) Breastfeeding; C) Serious bleeding disorder; D) Serious infections requiring antibiotics; E) Concomitant treatment with steroids or immunosuppressing agent; and F) Decision of unsuitableness by principal investigator or physician-in-charge.

### Treatment protocol

Each of the three peptides (1 mg each) was emulsified in 1 ml IFA, and injected into bilateral sites of the neck or inguinal at three separate regions. The vaccination was given subcutaneously once a week for five weeks as a 1 cycle and, after the 2 week interval, the next cycle was performed and continued as a monotherapy until the judgment of PD or doctor’s assessment. For the immunological evaluation, PBLs were obtained from the patients at the pre-vaccination period as well as after the 5^th^ and 10^th^ vaccinations. For the imaging analysis, CT scan was performed at pre-vaccination period (within 1 month before vaccination) and every 2 months after the vaccination.

### Peptides

Peptides derived from *TTK*-567 (SYRNEIAYL), *LY6K*-177 (RYCNLEGPPI), and *IMP3*-508 (KTVNELQNL) that bound to an HLA-A24 molecule were synthesized as described elsewhere [6]. The purity (>97%) of the peptides were determined by analytical high-performance liquid chromatography (HPLC) and mass spectrometry analysis, respectively. The endotoxin levels and bioburden of these peptides were tested and determined to be within acceptable levels as GMP grade for the vaccines (NeoMPS, Inc., San Diego).

### Lymphocyte preparation for immunologic monitoring

The performance of the immunological assay at the center laboratory was periodically standardized and validated by Clinical Laboratory Improvements Amendments (CLIA) and the International Conference on Harmonization of Technical Requirements for Registration of Pharmaceuticals for Human use (ICH) guideline [14,15].

PBLs were obtained from the patients at the pre-vaccination period and after the 5^th^ and 10^th^ vaccinations. Peripheral blood was taken by venipuncture, collected in EDTA tube and transferred to the center laboratory within 24 hrs at room temperature. Within 24 hrs from blood collection, PBLs were isolated with Ficoll-Paque Plus (GE Healthcare Bio-sciences, Piscataway, NJ) density gradient solution and were stored at −80 °C in cell stock media (Juji field) without serum at 5 x 10^6^ cells/ml. After thawing, cell viability was confirmed to be more than 90% by trypan-blue dye.

For the *in vitro* culture, PBLs were thawed simultaneously and 5 × 10^5^ cells per well were incubated in medium with 100 units/ml of recombinant IL-2 (rIL-2; Novartis) with the peptide stimulation (10 μg/ml) twice on day 1 and day 8 in combination with HIV-specific peptide (ILKEPVHGV, 10 μg/ml) as a negative control and CMV-specific peptide (RYLRDQQLL, 10 μg/ml) as a positive control. On day 15, the cultured lymphocytes were subjected to ELISPOT assay after depletion of CD4 cells by magnetic beads (Invitrogen, Grand Island, NY). For the 24(+) group, the conventional ELISPOT assay with TISI cells, which is human B-lymphoblastoid cells expressing HLA-A24 on their surface, pulsed with the relevant peptide as targets in combination with irrelevant HIV-specific peptide were performed, and followed by the HLA-A24 Pentamer assay. For the 24(−) group, modified ELISPOT with dump assay in the absence of APC as described below.

### ELISPOT assay

To monitor antigen-specific immune response, ELISPOT assay was performed with the human IFN-γ ELISPOT PLUS kit (Mabtech, Nacka Strand, Sweden). 96-well plates with nitrocellulose membranes (Millipore, Molshelm, France) were pre-coated with primary anti-IFN-γ antibody (1-D1K) at 4 °C overnight. The plates were then pre-reacted with RPMI medium containing 10% FBS (Invitrogen). For the 24 (+) group, each vaccine peptide (10 μg/ml)-, HIV-specific peptide (ILKEPVHGV, 10 μg/ml)- or CMV-specific peptide (RYLRDQQLL, 10 μg/ml)-pulsed TISI cells (2 × 10^4^/well) as stimulators were incubated for 24 hrs in triplicate with responder cells (from 2 × 10^4^/well to 2.5 × 10^3^/well) in a total of 200 μl/well in different responder/stimulator ratios as indicated. The stimulation with phorbol 12-myristate 13-acetate (PMA, 25 ng/mL, Sigma-Aldrich, St. Louis MO) + ionomycin (500 pM, Sigma-Aldrich) was used as a positive control for T cell activity. For the 24(−) group, responder cells (2 × 10^4^/well) and each peptide (10 μg/ml) or HIV-specific peptide (10 μg/ml) in a total of 200 μl/well were cultured without APC for 24 hrs in triplicate in combination with PMA + ionomycin as a positive control. These cell mixtures were treated with biotinylated secondary anti-IFN-γ antibody (7-B6-1) and incubated for 2 h. Then the plates were incubated with HRP-reagent and stained with TMB (Mabtech). The spots were quantified with an auto-analyzing system, ImmunoSPOT S4 (Cellular Technology Ltd). Positivity of antigen-specific T cell response was quantitatively defined according to our original evaluation tree algorithm (Additional file 1: Figure S1). In brief, the peptide-specific spots were the average of triplicates by subtracting the HIV peptide-pulsed stimulator well from the immunized peptide-pulsed stimulator well. The positivity of antigen-specific T cell response were classified into four grades (−, +, ++, and +++) depending on the amounts of peptide-specific spots and invariability of peptide-specific spots at different responder/stimulator ratios. When the algorithm indicated +, ++, or +++ at either 5^th^ or 10^th^ vaccination point, we judged to be positive case.

### Pentamer assay

For the 24(+) group, the *in vitro* cultured T cells were subjected to the pentamer assay to confirm the peptide-specificity. HLA-A24/TTK-, HLA-A24/LY6K-, or HLA-A24/IMP3-peptide pentamer (ProImmune) staining in the combination with CD8 and CD3 mAbs were performed and analyzed with flow cytometry.

### Statistical analysis

OS and PFS were analyzed by the Kaplan-Meier method, and the statistical differences were analyzed by Log-rank method. All statistical analyses were performed with SPSS statistics 17.0 (SPSS Inc.).

## Results

### Patient background

We recruited 60 eligible patients between October 1st, 2008 and April 3rd, 2010. The study database was locked on May 25, 2011. Expectedly, 58% of the enrolled patients possessed HLA-A*2402 (n = 35) and 42% of them were negative for HLA-A*2402 (n = 25) (Table 1) as similar to the reported allelic frequency in the Japanese population [13]. The backgrounds of the patients were not statistically different between the 24(+) and 24(−) groups as shown in Table 1. The analysis for all enrolled patients (n = 60) was performed by Intention-to-treat (ITT), although one patient in the A24 (+) group and one patient in the A24(−) group did not follow the treatment protocol perfectly.

**Table 1:** Patient background

	Total (n=60)	HLA-A24 (+) (n=35)	HLA A24 (-) (n=25)	
Age(year)	61.8±7.6	62.3±7.1	61.1±8.2	NS
Male: Female	53/7	32/3	21/4	NS
Prior therapy				
Operation	29(48%)	19(54%)	10(40%)	NS
Radiation	48(80%)	26(74%)	22(88%)	NS
Chemotheraphy				
5FU	59(98%)	35(100%)	24(96%)	NS
CDDP	58(97%)	33(94%)	23(92%)	NS
Docetaxel	54(90%)	31(91%)	23(92%)	NS

*NS* not significant between the HLA-A24(+) and HLA-A24(-) group.

### OS and PFS

**Figure 1:**
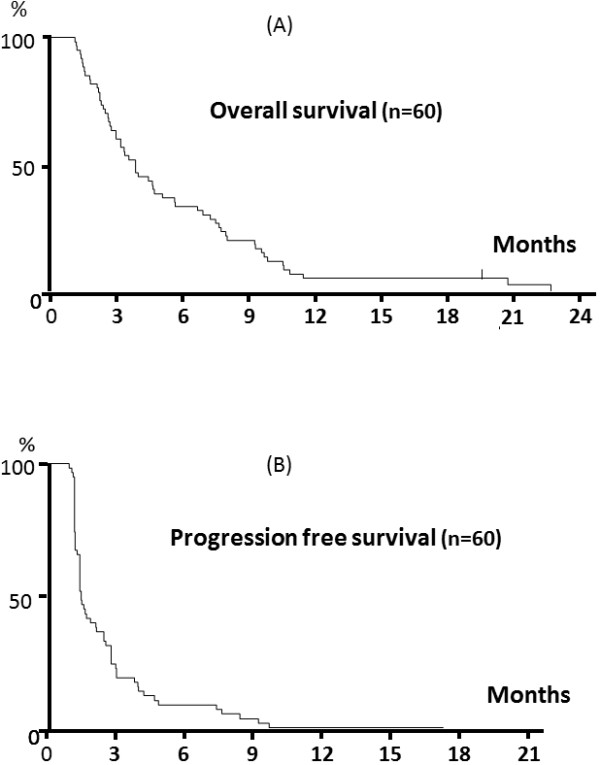
**Overall survival (OS) and progression free survival (PFS) in all enrolled patients.** The OS (**A**) and PFS (**B**) were analyzed by the Kaplan-Meier method. OS was measured in days from the 1st vaccination to death and the PFS were measured in days from the1st vaccination to disease progression.

**Figure 2:**
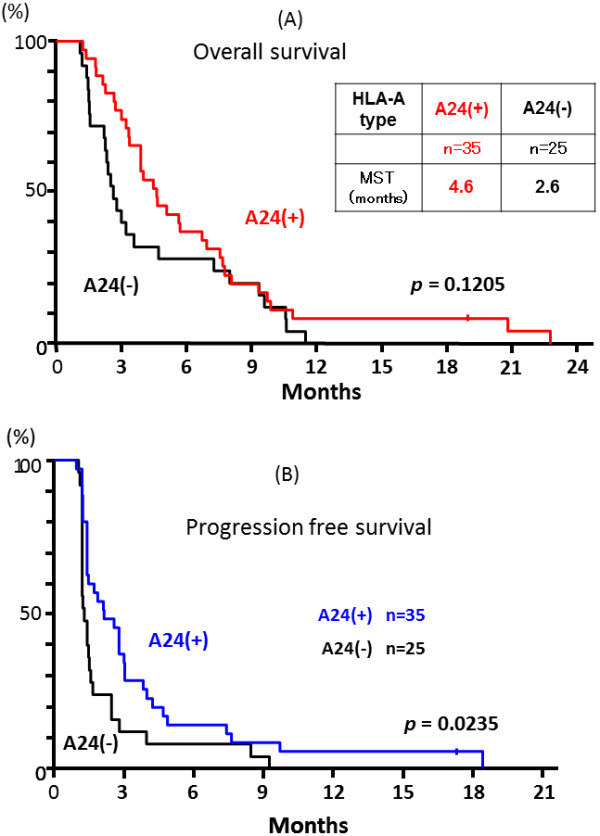
**Overall survival and progression free survival of the A24(+) and A24(−) groups.** All enrolled patients had received the vaccination without knowing HLA-A status, and the HLA-A genotypes were used for the key-open at analysis point. The OS (**A**) and PFS (**B**) were evaluated for each of the HLA-A*2402-positive (24(+)) and -negative (24(−)) groups for the sub-group analysis. MST, median survival time.

The OS and PFS in all enrolled patients after the vaccination were shown in Figure 1A and 1B. When the patients were classified into the 24(+) and 24 (−) groups, the OS in the 24(+) group tended to be better than that in the 24(−) group (4.6 vs. 2.6 month at MST, respectively, *p* = 0.121, Figure 2A), although the difference of OS was not statistically significant. However, the PFS in the 24(+) group was significantly better than that in the 24(−) group (*p* = 0.024, Figure 2B).

### OS in A24 (+) group related to Immunological monitoring

**Figure 3:**
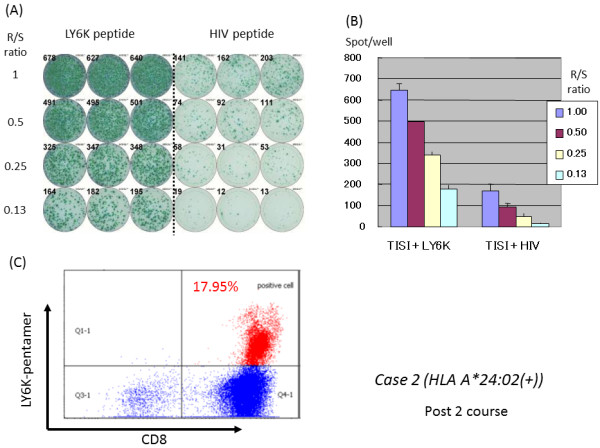
**Representative immunological monitoring assays detecting antigen-specific CTL response in a patient belonging to the 24(+) group.** PBLs obtained from case 2 patient (HLA-A*2402 positive) after the 10^th^ vaccination were cultured in rIL-2 for 14 days with 2 times of LY6K-peptide stimulation. (**A**) The cultured lymphocytes were subjected to the ELISPOT assay after depletion of CD4-positive cells by magnetic beads. TISI cells were incubated with responder cells in the presence of LY6K peptide or HIV peptide as an irrelevant control, and the spot counts were quantified (**B**). (**C**) The cultured lymphocytes were analyzed with HLA-A2402/LY6K-pentamer in the combination with CD8 and CD3 mAbs with flow cytometry. The value of pentamer (+)/CD8(+) among CD3(+) cells was shown. R/S, responder/stimulator.

For the A24 (+) group, *in vitro* cultured T cells were subjected to the ELISPOT and pentamer assays. Representative ELISPOT and pentamer assays specific to the LY6K peptide were shown in Figure 3. The ELISPOT assay indicated substantial T cell responses specific to the LY6K peptide in comparison to the irrelevant peptide (Figure 3A) according to the criteria described in Materials and Methods. This LY6K-specific T cell response was further confirmed by the LY6K-pentamer assay as shown in Figure 3C with the value of 17.95% of pentamer (+) + CD8(+) among CD3(+) cells.

In the 24(+) group, the positive CTL responses specific for the LY6K-, TTK-, and IMP3-peptide after the vaccination were observed in 63%, 45%, and 60% of the patients, respectively. Moreover, these peptide-specific T cells in the cultured T cells were all confirmed by the pentamer assays for the LY6K-, TTK-, and IMP3-peptide as 9.5-17.8%, 6.0–20.1%, and 5.1–15.5% of the positive cells, respectively in the value of pentamer (+) + CD8(+) among CD3(+) cells.

**Figure 4:**
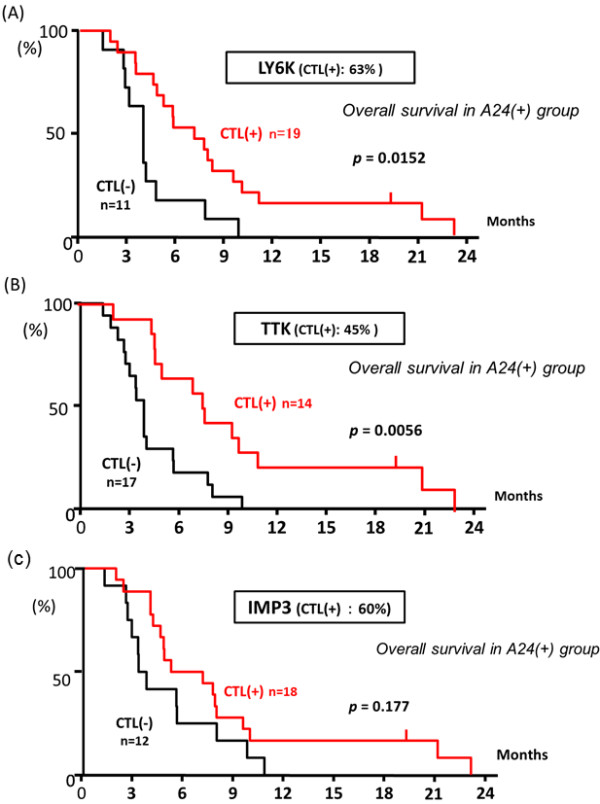
**OS in the A24 (+) group related to immunological monitoring specific to each of the LY6K, TTK, and IMP3 peptides.** In the 24(+) group, the *in vitro* cultured T cells were subjected to ELISPOT assays. The positive CTL responses specific to each of LY6K-, TTK-, and IMP3-peptides after the vaccination were observed in 63%, 45%, and 60% of the patients, respectively. The OS was compared between the patients with positive CTL response (+) and those with negative CTL response (−) for each peptide, the patients with positive CTL response specific to the LY6K-peptide revealed significantly better OS than those without CTL response (**A**). Similarly, the patients with positive CTL response specific to the TTK-peptide showed significantly better OS than those without CTL response (**B**). For IMP3, the patients with positive CTL response specific to the peptide tended to have better OS than those without CTL response, although the difference was not statistically significant (**C**).

**Figure 5:**
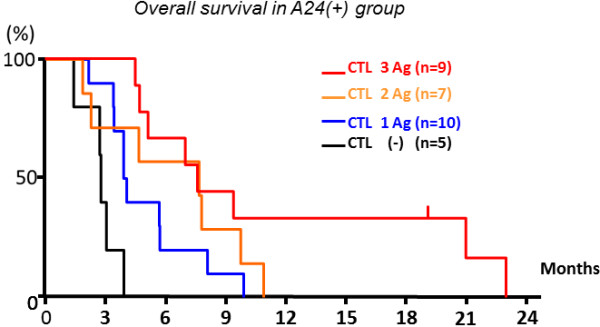
**OS in the four subgroups of the A24 (+) group classified by the number of the peptides showing the positive CTL responses.** The A24(+) patient group was classified into 4 groups according to the number of peptide antigens which induced the CTL responses (0, 1, 2 and 3). The OS tended to be better when the number of the peptides that induced CTL responses was higher.

When the OS was compared between the patients with CTL response (+) and (−) in the A24(+) group, the patients having CTL response specific to the LY6K peptide revealed significantly better OS than those without LY6K-specific CTL responses (Figure 4A). Similarly, the patients showing positive responses specific to the TTK peptide revealed significantly better OS than those without CTL responses (Figure 4B). OS of the patients with IMP3-specific CTL responses tended to be better than those without CTL responses, although the difference was not statistically significant (Figure 4C). Interestingly, when the patients were divided into 4 groups according to the number of antigens that they showed the positive responses, OS seemed to be better in the patient groups that showed the positive reaction to a larger number of the peptides (Figure 5); MST of the patients having CTL responses to all of three peptide was better (7.5 month) than those of other patient groups (Figure 5). Thus, these observations indicated that the immunological response induced by the peptide vaccination contribute to the improvement of prognosis of the patients.

### Immunological evaluation in the A24(−) group

*In vitro* cultured T cells from all patients of the A24 (−) group were subjected to the modified ELISPOT assay by applying the dump assay (the ELISPOT assay was performed without antigen presenting cells). A representative ELISPOT assay detected a presence of LY6K-peptide-specific T cells in *in vitro* cultured T cells (Additional file 2: Figure S2A) and their quantification experiment indicated positive CTL induction according to the criteria (Additional file 2: Figure S2B).

Through the modified ELISPOT assay, 3 (12%) of the 25 A24(−) patients were considered to have the peptide-specific CTL response against any of three antigens. Three patients with HLA-A*0201/-A*0301, HLA-A*0201/-A*0201 or HLA-A*0206/-A*3303 responded to the LY6K-peptide. This observation might reflect the cross-reactivity to a different HLA allele(s) against the vaccinated peptides in the present study. It might be notable that these three patients showed better OS in comparison to the patients with the ELISPOT-negative result in the 24(−) group (data not shown). Moreover, when the patients having the peptide-specific CTL response in the 24(−) group were excluded from the 24(−) group in the OS analysis, the OS in the 24(+) group (n = 35) were significantly better than those of the 22 patients in the 24(−) group (Additional file 2: Figure 2 C).

### Adverse reactions

The vaccination therapy was well-tolerated without any treatment-associated adverse events of grade 3 or higher. Thirty-two of the 60 patients developed grade 1 or 2 local skin reactions with redness and swelling in the injection sites. High grade fever, fatigue, diarrhea, headache, rash, and itching were not observed in any patients. No hematologic, cardiovascular, hepatic, or renal toxicity was observed during or after the vaccination.

## Discussion

The present phase II clinical study demonstrated that vaccination-induced immune response positively correlated with the better prognosis in advanced ESCC patients, implying that the cancer vaccine treatment with multi-epitope peptides as a monotherapy might provide the clinical benefit to the patients. To our knowledge, this study is the first to show a promising result indicating that therapeutic cancer vaccination with multiple peptides may improve the prognosis of patients with advanced solid tumors who had failed to standard therapy.

Several phase II and III clinical trials have recently demonstrated the promising and the therapeutic potentials of cancer vaccination [1-5,16-19]. However, most of them are performed with single antigen-based vaccination with several modifications and the clinical benefit seems to be very limited. In order to further improve the clinical responses of cancer vaccination treatment, it is necessary to consider the application of a combination of multiple vaccines derived from the different target molecules, because it may overcome the issue of heterogeneity of tumor cells and also avoid the escape of tumor cells from peptide-specific immune response by loss of antigen expression [5,20]. In general, the preferable characteristic of the target molecules for development of cancer vaccines are (1) high immunogenicity, (2) very common expression in cancer cells, (3) specific expression in cancer cells and (4) essential molecules for cell survival (to avoid loss of expression) [5,20]. In this regard, TTK, LY6K, and IMP3 molecules used in the present trials are considered to be most appropriate because they were expressed in the great majority (> 95%) of esophageal cancers, were expressed specifically in cancer cells and testis (cancer-testis antigens), were shown to be essential for survival of cancer cells, and most importantly revealed very strong immunogenicity [6-12].

In the present phase II clinical trial, we compared the OS and PFS between the A24(+) and A24(−) groups classified by the HLA-A*2402 positivity as a biological marker. The PFS and, less significantly, OS in the A24(+) group were better than those in the A24(−) group, suggesting that the therapeutic cancer vaccine treatment using the HLA-A24-restricted peptides could induce some survival benefits for advanced ESCC patients. Furthermore, in the A24 (+) group, the specific CTL responses to multiple peptides may improve OS in comparison with the cases with the CTL induction to no or a single peptide. Although treatment with cancer vaccines had been shown to cause an increase of circulating tumor antigen-specific T cells [21], we have here demonstrated the direct evidence of positive correlation between the extent of peptide-specific CTL responses and better OS. Hence, our present study supports the concept that vaccination-induced immune response could contribute to the improvement of prognosis of advanced ESCC patients.

According to the recommendation from the iSBSTc-SITC/FDA/NCI Workshop on Immunotherapy Biomarkers [22-24], we have performed the immunological monitoring in the A24 (+) group by two different assays, ELISPOT and pentamer assays, at three different time points and at the center laboratory. Since the peptides used in the present study had strong immunogenicity, the *in vitro* immunological monitoring in the A24 (+) group was successfully performed in a reliable way.

In the 24(−) group, the modified ELISPOT assay without APC suggested that the cross-reactivity with the different HLA allele(s) against the vaccinated peptides may exist in some extent. Although the modified ELISPOT assay in the 24(−) group was not fully standardized due to lack of APC and appropriate positive controls, we suspect that this cross-reactivity in patients in the 24(−) group might influence the clinical outcome. Three patients with positive ELISPOT assays did not have a common HLA-A allele genotype, but two of them had HLA-A*0201 and the other had HLA-A*0206 that is known to structurally similar to HLA-A*0201. Although the LY6K-peptide used in the present clinical trial is predicted to have a weak-binding affinity to HLA-A0201 by BIMAS and NetMHC programs, the peptide might cross-react to HLA-A0201 and their structurally similar HLA*.* Further study to clarify whether the LY6K-peptide could actually bind to HLA-A0201 and the other HLA molecules, and whether the immunogenicity in context with HLA-A0201 is confirmed by CTLs will be needed.

Since available cancer vaccines probably are likely to be applied as adjuvant treatment for the patients who are at high risk of relapse following surgical resection of primary tumor [5,20], and we are planning to develop these cancer vaccine as an adjuvant setting for ESCC patients with a lymph node metastasis after curative esophagectomy. However, even if the patients are at an advanced stage and have been intensively treated with chemotherapy and/or radiotherapy, the present study indicated that cancer vaccine may provide some clinical benefit as a monotherapy without any severe side effects. In general, there is no curative therapy for ESCC patients with in-operable tumor or those with recurrence after surgery. Platinum-based regimens including 5FU, CDDP and Taxans have been used as the first line chemotherapy for these patients, but the median survival time of such patients was as short as 7–8 months [25,26]. However, there is no effective treatment available for patients with ESCC that are refractory to the first line chemotherapy. Hence, we are confident that our protocol should be promising for improvement of the prognosis and quality of life even for advanced ESCC patients.

## Conclusions

To our knowledge, this study is the first to show the proof of concept that vaccination-induced immune response could provide the better prognosis in advanced ESCC patients and has implied that cancer vaccine treatment with multiple peptides as monotherapy can be a hope to patients with advanced solid tumors who had failed to standard therapy.

## Competing interests

Koji Yoshida and Takuya Tsunoda are current employees of Oncotherapy Science, Inc. Yusuke Nakamura is a stockholder of Oncotherapy Science, Inc.

## Authors’ contributions

KK, HI, YA, HT, NH, YU, TN, and KO participated in collection and assembly of data. KT, KY, and TT participated in the Immunological assay. All authors participated in the design of the study and performed the statistical analysis. KK, KY and YN participated in manuscript writing. All authors read and approved the final manuscript.

## Supplementary Material

Additional file 1 **Figure S1.** Positivity of antigen-specific T cell response was quantitatively defined according to the evaluation tree algorithm. In brief, the peptide-specific spots (SS) were the average of triplicates by subtracting the HIV peptide-pulsed stimulator well from the immunized peptide-pulsed stimulator well. The %SS means the percentage of SS among the average spots of the immunized peptide-pulsed stimulator well. The positivity of antigen-specific T cell response were classified into four grades (−, +, ++, and +++) depending on the amounts of peptide-specific spots and invariability of peptide-specific spots at different responder/stimulator ratios. For example, the ELISPOT data from Figure 3 (SS = 480 at ratio 1, SS = 404 at ratio 0.5, SS = 292 at ratio 0.25, and %SS = 74 at ratio 1, %SS = 88 at ratio 0.5) matched to the criteria (1) and (2) in the first step and then, the data matched to criteria (1) in the second step. Thus, the ELISPOT data was finally evaluated as (+++). SS, peptide-specific spots; R1, responder/stimulator ratio = 1; R2, responder/stimulator ratio = 0.5; R3, responder/stimulator ratio = 0.25; R4, responder/stimulator ratio = 0.125. Click here for file

Additional file 2 **Figure S2.** Immunological evaluation in the A24(−) group. The *in vitro* cultured T cells from all patients of the A24 (−) group were subjected to the modified ELISPOT assay by applying the dump assay (the ELISPOT assay was performed without antigen presenting cells). A representative ELISPOT assay detected a presence of LY6K-peptide-specific T cells in *in vitro* cultured T cells (Additional file 2: Figure S2A) and their quantification experiment indicated positive CTL induction according to the criteria (Additional file 2: Figure S2B). Through the modified ELISPOT assay, 3 (12%) of the 25 A24(−) patients were considered to have the peptide-specific CTL response against any of three antigens. When the patients having the peptide-specific CTL response in the 24(−) group were excluded from the 24(−) group in the OS analysis, the OS in the 24(+) group (n = 35) were significantly better than those of the 22 patients in the 24(−) group (Additional file 2: Figure S2C). Click here for file
