# Supplementary material for: Multicenter, phase II clinical trial of cancer vaccination for advanced esophageal cancer with three peptides derived from novel cancer-testis antigens
Source: J Transl Med. 2012 Jul 9;10:141. doi: 10.1186/1479-5876-10-141 (PMC3403921; doi:10.1186/1479-5876-10-141)
Supplement: Additional file 1 — Figure S1. Positivity of antigen-specific T cell response was quantitatively defined according to the evaluation tree algorithm. In brief, the peptide-specific spots (SS) were the average of triplicates by subtracting the HIV peptide-pulsed stimulator well from the immunized peptide-pulsed stimulator well. The %SS means the percentage of SS among the average spots of the immunized peptide-pulsed stimulator well. The positivity of antigen-specific T cell response were classified into four grades (−, +, ++, and +++) depending on the amounts of peptide-specific spots and invariability of peptide-specific spots at different responder/stimulator ratios. For example, the ELISPOT data from Figure 3 (SS = 480 at ratio 1, SS = 404 at ratio 0.5, SS = 292 at ratio 0.25, and %SS = 74 at ratio 1, %SS = 88 at ratio 0.5) matched to the criteria (1) and (2) in the first step and then, the data matched to criteria (1) in the second step. Thus, the ELISPOT data was finally evaluated as (+++). SS, peptide-specific spots; R1, responder/stimulator ratio = 1; R2, responder/stimulator ratio = 0.5; R3, responder/stimulator ratio = 0.25; R4, responder/stimulator ratio = 0.125. [file 1479-5876-10-141-S1.ppt]

## Slide 1
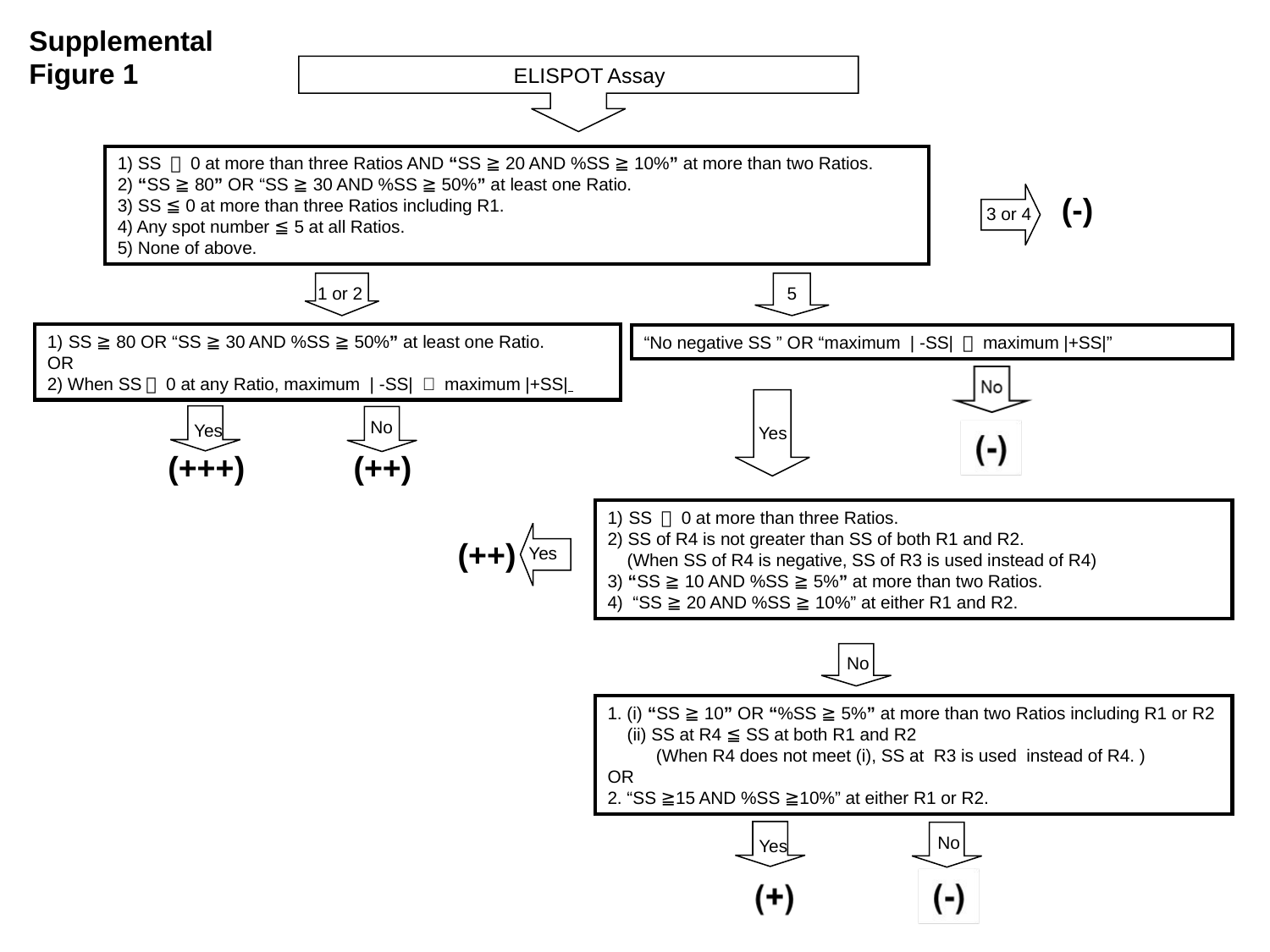

Supplemental
Figure 1
ELISPOT Assay
1) SS ＞ 0 at more than three Ratios AND “SS ≧ 20 AND %SS ≧ 10%” at more than two Ratios.
2) “SS ≧ 80” OR “SS ≧ 30 AND %SS ≧ 50%” at least one Ratio.
3) SS ≦ 0 at more than three Ratios including R1.
4) Any spot number ≦ 5 at all Ratios.
5) None of above.
(-)
3 or 4
1 or 2
5
SS ≧ 80 OR “SS ≧ 30 AND %SS ≧ 50%” at least one Ratio.
OR
2) When SS＜ 0 at any Ratio, maximum | -SS| ＜ maximum |+SS|
“No negative SS ” OR “maximum | -SS| ＜ maximum |+SS|”
No
Yes
Yes
(+++)
(++)
SS ＞ 0 at more than three Ratios.
2) SS of R4 is not greater than SS of both R1 and R2.
 (When SS of R4 is negative, SS of R3 is used instead of R4)
3) “SS ≧ 10 AND %SS ≧ 5%” at more than two Ratios.
4) “SS ≧ 20 AND %SS ≧ 10%” at either R1 and R2.
(++)
Yes
No
1. (i) “SS ≧ 10” OR “%SS ≧ 5%” at more than two Ratios including R1 or R2
 (ii) SS at R4 ≦ SS at both R1 and R2
 (When R4 does not meet (i), SS at R3 is used instead of R4. )
OR
2. “SS ≧15 AND %SS ≧10%” at either R1 or R2.
No
Yes
