# Supplementary material for: Multicenter, phase II clinical trial of cancer vaccination for advanced esophageal cancer with three peptides derived from novel cancer-testis antigens
Source: J Transl Med. 2012 Jul 9;10:141. doi: 10.1186/1479-5876-10-141 (PMC3403921; doi:10.1186/1479-5876-10-141)
Supplement: Additional file 2 — Figure S2. Immunological evaluation in the A24(−) group. The in vitro cultured T cells from all patients of the A24 (−) group were subjected to the modified ELISPOT assay by applying the dump assay (the ELISPOT assay was performed without antigen presenting cells). A representative ELISPOT assay detected a presence of LY6K-peptide-specific T cells in in vitro cultured T cells (Additional file 2: Figure S2A) and their quantification experiment indicated positive CTL induction according to the criteria (Additional file 2: Figure S2B). Through the modified ELISPOT assay, 3 (12%) of the 25 A24(−) patients were considered to have the peptide-specific CTL response against any of three antigens. When the patients having the peptide-specific CTL response in the 24(−) group were excluded from the 24(−) group in the OS analysis, the OS in the 24(+) group (n = 35) were significantly better than those of the 22 patients in the 24(−) group (Additional file 2: Figure S2C). [file 1479-5876-10-141-S2.pptx]

## Slide 1
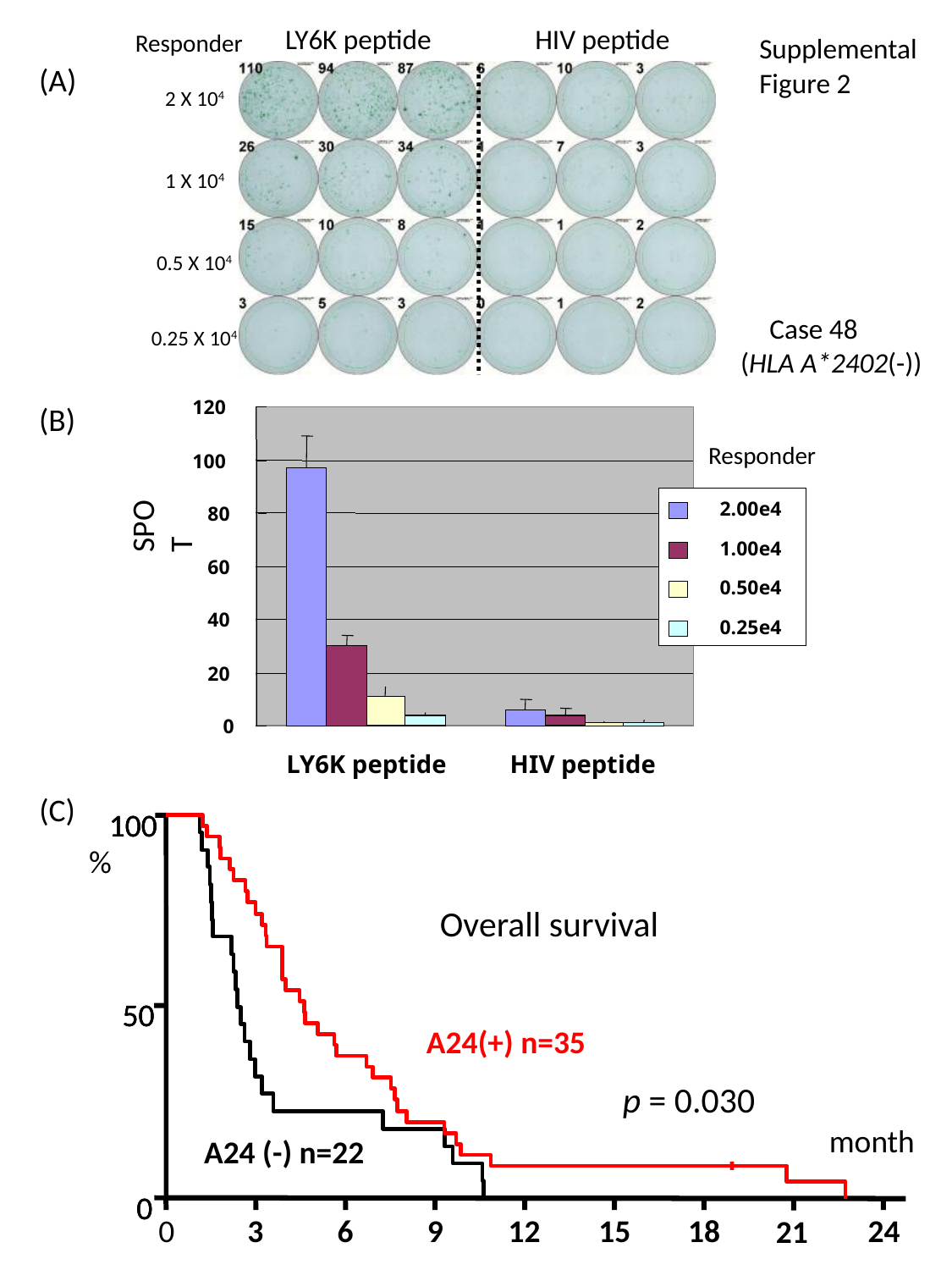

LY6K peptide
HIV peptide
Responder
2 X 104
1 X 104
0.5 X 104
0.25 X 104
Supplemental
Figure 2
(A)
Case 48
(HLA A*2402(-))
(B)
120
100
2.00e4
80
1.00e4
60
0.50e4
40
0.25e4
20
0
LY6K peptide HIV peptide
Responder
SPOT
(C)
100
100
100
50
50
50
0
0
0
0
3
6
9
12
15
18
24
21
A24(+) n=35
p = 0.030
A24 (-) n=22
%
Overall survival
month
